# Successful endovascular repair with coil embolization of ruptured right internal thoracic artery aneurysm

**DOI:** 10.1590/1677-5449.20210223

**Published:** 2022-07-11

**Authors:** Yamasandi Siddegowda Shrimanth, Atit A. Gawalkar, Parag Barwad, Soumitra Ghosh, Samman Verma, Arun Sharma, Sanjeev Naganur

**Affiliations:** 1 Post Graduate Institute of Medical Education & Research – PGIMER, Chandigarh, India.

**Keywords:** internal thoracic artery aneurysm, aneurysm rupture, coil embolization, endovascular repair, CT angiography, percutaneous repair, aneurisma da artéria torácica interna, ruptura do aneurisma, embolização com molas, reparo endovascular, angiotomografia, reparo percutâneo

## Abstract

Internal thoracic artery aneurysms (ITAAs) are rare with wide variation in clinical presentation and a high risk of rupture. Endovascular techniques are increasingly being used for treatment of such aneurysms over surgical repair in recent times. A 34-year-old male presented with progressive swelling of the right anterior chest wall for 2 weeks and was diagnosed with right internal thoracic artery aneurysm with contained rupture. He underwent successful endovascular repair with coil embolization of ruptured right ITAA. Post intervention computed tomography (CT) angiography confirmed sealing of the ruptured aneurysm with no residual filling of the sac. At six months follow-up he is doing well with complete resolution of hematoma. This case demonstrates that an endovascular approach with coil embolization is a feasible and safe option for treating the rare ruptured ITAAs.

## Resumo

Os aneurismas da artéria torácica interna (ITAAs) são raros, com ampla variação na apresentação clínica e alto risco de ruptura. As técnicas endovasculares têm sido cada vez mais utilizadas para o tratamento desses aneurismas em relação ao reparo cirúrgico. Um homem de 34 anos de idade apresentou edema progressivo da parede torácica anterior direita por 2 semanas e foi diagnosticado com aneurisma da artéria mamária interna direita com ruptura contida. Ele foi submetido a reparo endovascular bem-sucedido, com embolização de ITAA direito roto. A angiotomografia computadorizada (angioTC) pós-intervenção confirmou o selamento do aneurisma rompido, sem enchimento residual do saco. No seguimento de 6 meses, ele estava bem, com resolução completa do hematoma. Este caso demonstra que a abordagem endovascular com embolização com molas é uma opção viável e segura no tratamento dos raros ITAAs rotos.

## INTRODUCTION

Internal thoracic artery aneurysm (ITAA) is a rare clinical entity that usually presents as a pseudoaneurysm following sternotomy, complicated pacemaker implantation or central venous catheter placement, or endovascular procedures.^1^ Rupture of an ITAA can be life threatening. It has been traditionally treated with aneurysmectomy, however endovascular techniques have been used successfully.^1^^-^ 7 We report a case of ruptured right internal thoracic artery (RITA) aneurysm presenting as right anterior chest wall bulge, which was successfully treated with endovascular coil embolization.

Informed written consent has been obtained by the patient for publication of images and clinical details.

## CASE PRESENTATION

**Figure 1:**
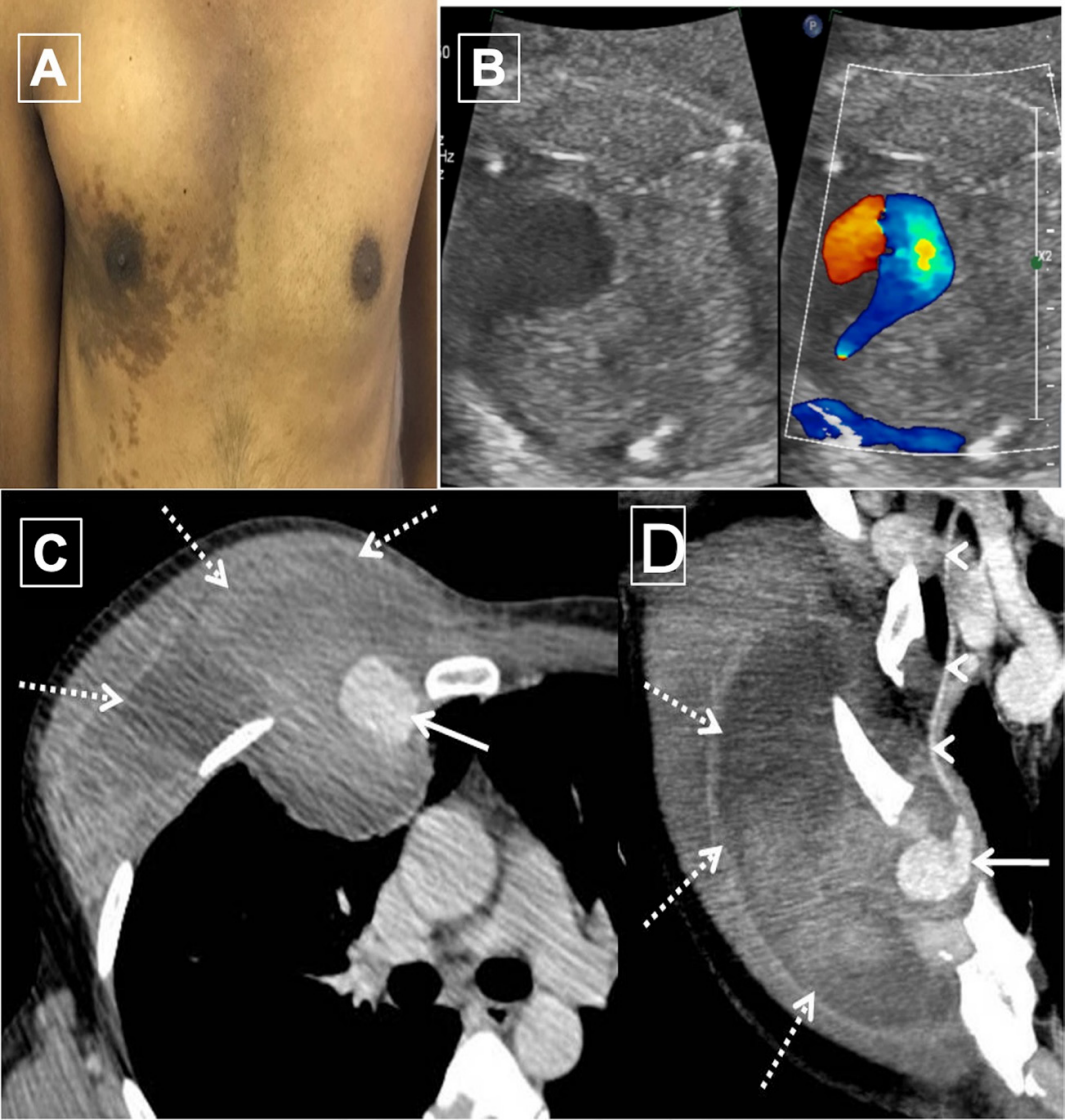
(A) Clinical photograph showing bulge in the right mammary and infraclavicular areas; (B) Right internal thoracic artery (RITA) aneurysm showing bidirectional blood flow with characteristic yin yang sign; (C and D) Pre-coiling CT: CT angiography images show presence of ruptured aneurysm sac (arrow) arising from the RITA (arrowheads) with surrounding large hematoma (dashed arrows) causing contour bulge along anterior chest wall on right side.

A 34-year-old gentleman presented with progressive swelling of the right anterior chest wall for 2 weeks. He denied any trauma or fever. Examination revealed an ill-defined soft tender swelling arising from the intermuscular plane in the right infraclavicular and mammary region (Figure 1A). Doppler ultrasound showed a large hyperechoic area with eccentric hypoechoic region and bidirectional flow with the characteristic Yin-Yang sign (Figure 1B) along the right anterior chest wall. Computed tomography angiography (CTA) (Figure 1C) showed presence of an aneurysm arising from the RITA, surrounded by large hematoma suggestive of contained rupture. He was transfused with two units of packed red cells for severe anemia (hemoglobin 6g/dl) and was taken up for coil embolization.

**Figure 2:**
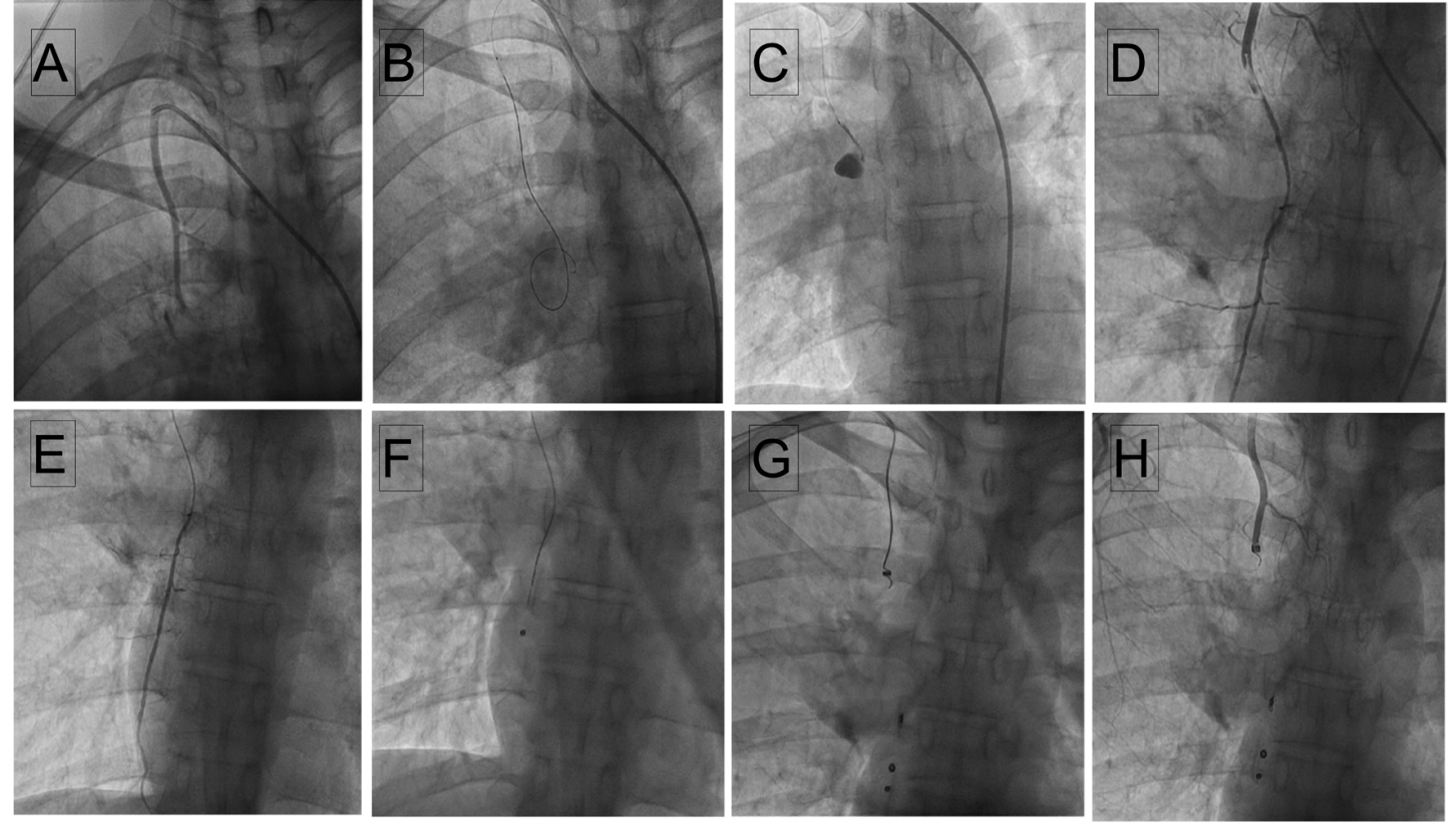
Selective RITA injection from RFA access (A) Coronary 018 wire into the aneurysm (B) Microcatheter injection showing microleak (C) Wire/microcatheter induced dissection in the distal part of the RITA (D) Coronary wire parked in the right superior epigastric artery from right brachial access (E) Injection through microcatheter showing flow in the right superior epigastric artery (F) Micro coils deployed distal and proximal to the aneurysm (G and H) Selective RITA injection demonstrating RITA occlusion (I).

**Figure 3:**
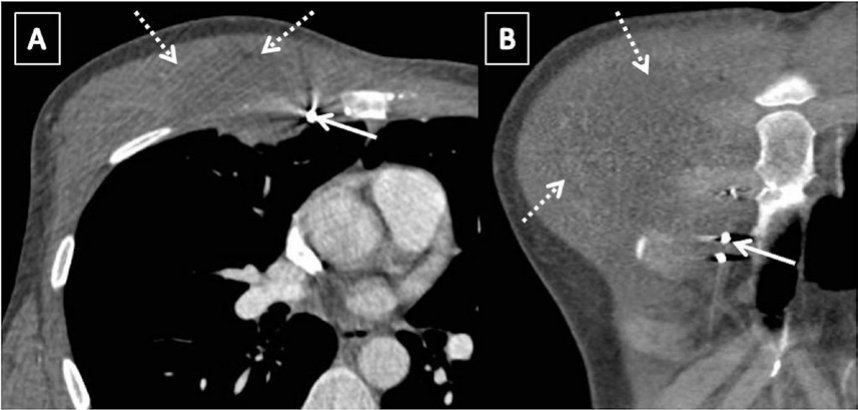
Post-coiling CT: CT images (A and B) following coil embolization show no residual filling of the sac with coil mass in situ (arrow) and resolving residual hematoma (dashed arrows).

Selective injection of the RITA with a Judkins right diagnostic catheter from right femoral access showed leakage from the distal part of the RITA (Figure 2A). The aneurysm and leak were delineated with an injection into the microcatheter which was traversed over a 0.018-coronary guidewire (Figure 22C). As a dissection was noted in the RITA proximal to the aneurysm during manipulation of catheter and wires (Figure 2D), we shifted to brachial access for ease of hardware manipulation. A 0.018 Coronary wire was parked in the right superior epigastric artery and a microcatheter was traversed over it (Figure 2E). Through the microcatheter, multiple micro coils (HILAL 2.0-2-18, HILAL 3.0-3-18, NESTER 18-5-5, all COOK MEDICALS) were embolized distal and proximal to the aneurysm (Figure 22G). Final selective RITA injection demonstrated RITA occlusion with insignificant flow of blood into the aneurysm (Figure 2H). Post intervention CTA confirmed sealing of the ruptured aneurysm with no residual filling of the sac (Figure 3) with resolving residual hematoma. At 6 months follow up, the patient is fine with no clinical suspicion of aneurysm of any of peripheral arteries.

## DISCUSSION

ITAA is rare and is usually associated with trauma, collagen vascular diseases, post sternotomy, central venous catheter placement, and chest wall infections.^2^^,^ 8 There are only a handful of case reports of idiopathic ITAA, associated with atherosclerosis, fibromuscular dysplasia, or cystic medial degeneration.^8^ Consequently, there are no standard guidelines for diagnosing and managing the same.

It usually presents with cough, dyspnea, hemoptysis, chest pain, and, rarely, with continuous murmurs, painful chest wall edema, accompanied by a bulging chest mass.^6^ It may rupture and present catastrophically with hemomediastinum or hemothorax.^6^ Interestingly, up to 50% of aneurysms may be discovered incidentally as a hilar or mediastinal mass by radiology.^1^ A chest X ray (CXR) may show an aneurysm even in the absence of clinical findings, but CT angiography or invasive/conventional angiography is essential to delineate the aneurysm and formulate a treatment plan.^4^

Due to rapid growth, ITAAs are prone to rupture.^3^ 37% of cases have presented as ruptured aneurysms, and even small aneurysms have been reported to rupture.^3^ Therefore, early diagnosis and treatment are imperative. Treatment includes endovascular and open surgical modalities. Open surgical repair may be preferred in some instances including large aneurysms with compressive symptoms and cases requiring a biopsy,^4^ but it is associated with complications like bleeding, surgical site infection, risks of general anesthesia, and longer duration of hospital stay.^6^ Endovascular therapy is especially effective for patients with high surgical risk and for patients with connective tissue disorders.^8^ Endovascular embolization can be achieved using coils, polymer, or glue.^5^ The aneurysm can also be excluded using covered stents.^7^ Complications of endovascular therapy include reflux via collaterals leading to enlargement of the aneurysm, and recurrence post-embolization. Nonetheless, the success rate of embolization in ITAAs has been reported to be 94.3%.^6^ Follow-up imaging of the patient is not available and is a limitation of this case report.

## CONCLUSIONS

We describe an extremely rare case of isolated, ruptured RITA aneurysm presenting with intermuscular hematoma. It was successfully treated by endovascular coil embolization.
